# MIDDLE-AGED ADULTS’ BEREAVEMENT RESPONSE TO PARENT DEATH: THE ROLE OF PRE-LOSS RELATIONSHIP QUALITY

**DOI:** 10.1093/geroni/igz038.2250

**Published:** 2019-11-08

**Authors:** Kyungmin Kim, Jeffrey E Stokes, Steven H Zarit, Karen L Fingerman

**Affiliations:** 1 University of Massachusetts Boston, Boston, Massachusetts, United States; 2 Pennsylvania State University, University Park, Pennsylvania, United States; 3 The University of Texas at Austin, Austin, Texas, United States

## Abstract

The bereavement literature in adulthood has largely focused on spousal loss. Yet the death of a parent is an influential – and expected – loss experience in middle and later life. This study analyzed prospective data from two waves of the Family Exchanges Study (2008 and 2013) to explore adults’ (N = 192; Mage = 56.76) experience of a recent parent death in the past 5 years, including grief responses and positive memories of the deceased parent. We examined how pre-loss relationships with the deceased parent (e.g., positive and negative relationship quality, relationship importance) are associated with different bereavement responses among the bereaved children. Findings showed that the levels of grief were higher for children who placed more importance on the parent prior to that parent’s death. Positive relationship quality was associated with positive memories after a parent’s death. However, negative relationship quality was not associated with any bereavement responses.

